# Response of SI cortex to ipsilateral, contralateral and bilateral flutter stimulation in the cat

**DOI:** 10.1186/1471-2202-6-29

**Published:** 2005-04-22

**Authors:** Mark Tommerdahl, Stephen B Simons, Joannellyn S Chiu, Oleg Favorov, Barry Whitsel

**Affiliations:** 1 Department of Biomedical Engineering, University of North Carolina, Chapel Hill, NC 27599, USA; 2 Department of Cellular and Molecular Physiology, University of North Carolina, Chapel Hill, NC 27599, USA

## Abstract

**Background:**

While SII cortex is considered to be the first cortical stage of the pathway that integrates tactile information arising from both sides of the body, SI cortex is generally not considered as a region in which neuronal response is modulated by simultaneous stimulation of bilateral (and mirror-image) skin sites.

**Results:**

Optical intrinsic signal imaging was used to evaluate the response of SI and SII in the same hemisphere to 25 Hz sinusoidal vertical skin displacement stimulation ("skin flutter") applied contralaterally, ipsilaterally, and bilaterally (simultaneously) to the central pads of the forepaws. A localized increase in absorbance in both SI and SII occurred in response to both contralateral and bilateral flutter stimulation. Ipsilateral flutter stimulation evoked a localized increase in absorbance in SII, but little or no change in SI absorbance. In the forepaw representational region of SI, however, bilateral stimulation of the central pads evoked a response substantially smaller (approximately 30–35% smaller) than the response to flutter stimulation of the contralateral central pad.

**Conclusion:**

The finding that the response of SI cortex to bilateral central pad flutter stimulation is substantially smaller than the response evoked by a contralateral flutter stimulus, together with the recently published observation that a region located posteriorly in SII responds with a substantially larger response to a bilateral flutter stimulus than the response evoked from the contralateral central pad, lead us to propose that the SI activity evoked by contralateral skin stimulation is suppressed/inhibited (via corticocortical connections between SII and SI in the same hemisphere) by the activity a simultaneous ipsilateral skin stimulus evokes in posterior SII.

## Background

It is established that multiple fields/areas in each cerebral hemisphere are activated at short latency by stimuli that trigger spike discharge activity in skin mechanoreceptive afferents. Of these the most extensively studied is SI – a region which most investigators have come to regard as responsive solely (the major exception being the face region of SI) to tactile stimuli delivered to contralateral skin sites. In contrast, SII has long been known to be activated at short latency by mechanical stimulation of skin sites on both sides of the body midline. Although this differential responsivity of SI and SII to stimulation of contralateral *vs. *ipsilateral skin sites has been widely accepted for more than five decades (since the pioneering evoked potential mapping studies of C.N. Woolsey and colleagues; e.g. [1]), the contribution of SII activation to cortical tactile information processing remains unknown. Additionally, although SII is considered to be the first cortical stage of the pathway that integrates information arising from both sides of the body, SI is generally not considered as a cortical region in which ipsilateral inputs play a major role in bilateral integration of information across the body mid-line. It is known, however, that even the distal limb representational regions in SI receive axonal projections (via the corpus callosum) from SI neurons in the opposite hemisphere [2,3].

Recently, we reported the results from experiments in which we obtained simultaneous observations of the activity evoked in both SI and SII in the same hemisphere of cat cerebral cortex by a 25 Hz sinusoidal vertical skin displacement stimulus ("skin flutter") applied contralaterally, ipsilaterally, or bilaterally to the central pads of the forepaws [4]. Briefly summarized, a localized increase in absorbance in both SI and SII was evoked by contralateral and also by bilateral flutter stimulation. Ipsilateral flutter stimulation also evoked a localized increase in absorbance in SII, but only a weak or negligible increase in the forepaw region of SI. Interestingly, the region of SII that responded with an increase in absorbance to ipsilateral stimulation was 2–3 mm posterior to the region in which absorbance increased maximally in response to stimulation of the contralateral central pad. Furthermore, in the posterior SII region that yielded the largest response to ipsilateral stimulation of the central pad, the response to bilateral central pad stimulation approximated a linear summation of the SII responses to independent stimulation of the contralateral and ipsilateral central pads. Conversely, in the anterior region of SII (the region that exhibited the largest response to contralateral stimulation), the response to bilateral stimulation was consistently smaller than (by approximately 30–35%) the response evoked from the contralateral central pad.

This report addresses the response of SI cortex to the same modes of stimulation used in the above-described study that focused on SII (i.e., contralateral, ipsilateral, and bilateral stimulation of the central pads of the forepaws). The central finding is that flutter stimulation of the ipsilateral central pad exerts a suppressive/inhibitory influence on the SI response to a 25 Hz flutter stimulus to the contralateral central pad (forepaw). In addition, the temporal relationship between stimulus-evoked activity in selected locations in the responding regions of SI and SII is evaluated quantitatively (using the approach of correlation mapping), revealing a previously unrecognized, and presumably functionally important high degree of coordination between the activities evoked by forepaw stimulation in both SI and the recently identified [4] anterior *vs. *posterior components that comprise SII cortex in the same hemisphere.

## Results

**Figure 1:**
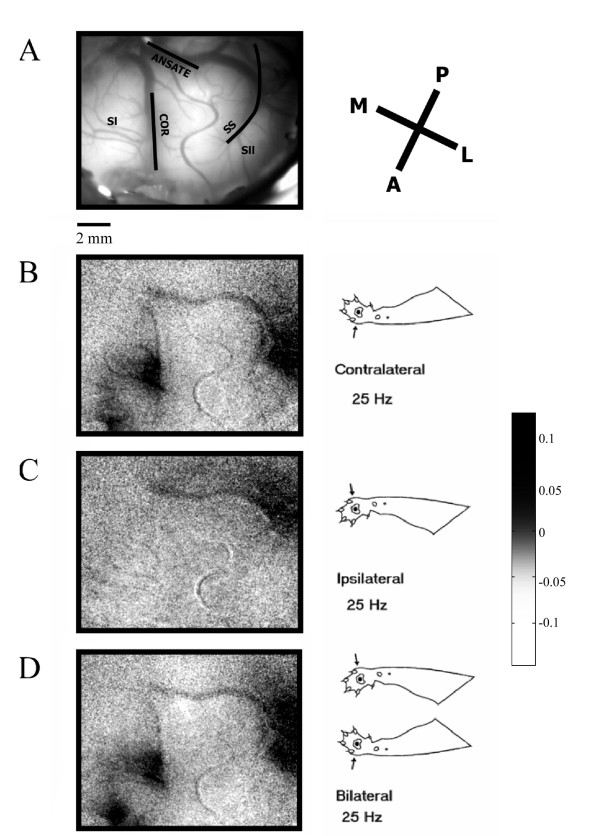
Cat SI and SII optical responses to 25 Hz vibrotactile stimulation of the forepaws. A. View of the cortical surface, showing the vascular pattern and coronal (COR), ansate (ANS), and suprasylvian (SS) sulci. Exposed portions of SI and SII are indicated. *Below*: Averaged absorbance images for responses evoked by (B) contralateral, (C) ipsilateral and (D) bilateral stimuli. Individual absorbance images were generated by subtracting each prestimulus (reference) image from its corresponding poststimulus image and subsequently dividing by the reference image. Averages are generated by summating the data obtained at a particular frame across trials (in this case, at 5 sec after stimulus onset). Stimulus sites are indicated by figurines. Scale bar is 2 mm. Orientation of images indicated by P (posterior), A (anterior), M (medial) and L (lateral) axes. Components of this figure have been previously reported [4].

Figure 1 shows the optical (OIS) responses evoked in SI and SII in one exemplary subject by contralateral, ipsilateral, and bilateral central pad stimulation. Visual inspection of the images for the three stimulus conditions shows that: (1) the SI response to bilateral stimulation occupies the same region that responded to stimulation of the contralateral central pad; and (2) stimulation of the ipsilateral central pad evokes little or no absorbance change in SI. Results such as those shown in Figure 1 were consistent across all subjects studied (n = 5) and have been reported previously [4]. In all subjects, the area of SI that responded with an increase in absorbance was confined to areas 3b/1. The region of area 2 that corresponds to the central pad of the forepaw is normally buried in the anterior bank of the ansate sulcus and could not be imaged. Inadvertently, the position of the cortical recording chamber, and thus the region of pericoronal cortex that was imaged, varied from one subject to the next. In Figure 1, for example, a region anterior to SI (bottom left corner; identified cytoarchitectually as area 4γ) was included in the imaged field and interestingly, this region not only responded vigorously to contralateral stimulation, but the magnitude of the absorbance increase in this area was largest with bilateral stimulation. Unfortunately, area 4γ was not imaged in any of the other subjects.

**Figure 2:**
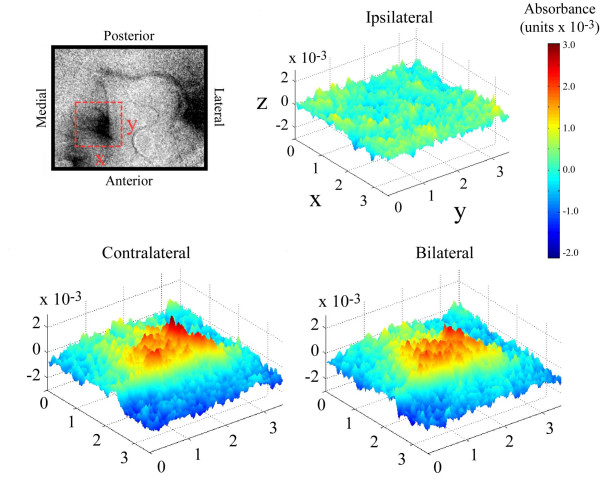
Surface plots of absorbance evoked in SI by contralateral, ipsilateral, and bilateral stimulation. Data displayed is a subset of the data displayed in Figure 1. Region of interest is indicated by the dashed box shown in the OIS image (top left). Orientation of the selected region is indicated by X (medial-lateral) and Y (anterior-posterior) labels on the dashed box and axes (in ipsilateral map). Cortical space along X and Y axes is measured in mm. Z axis is absorbance which is represented by both the height of the graphic as well as the color indicated by the color bar to the right. Surface plots represent absorbance values within the ROI at 5 seconds after stimulus onset and demonstrate that 1)response in SI to ipsilateral stimulation is small or nonexistent and, 2)magnitude of evoked absorbance in response to contralateral stimulation is slightly larger than the response evoked by bilateral stimulation.

The SI response to each of the different stimulus conditions can be better appreciated when the imaging data are displayed as a multi-dimensional surface plot. Figure 2 compares the stimulus evoked response of SI to contralateral *vs. *bilateral stimulation at 5 seconds after stimulus onset (stimulus duration always was 5 seconds). The top left panel indicates the orientation of the sampled region of interest (ROI) in SI. The surface plots show the absorbance value at each pixel location in the imaged field, with absorbance defined both by elevation along the z-axis and by pseudocolor (indicated by the color-coded scale bar at right). Consistent with the difference images shown in Figure 1, the response evoked by ipsilateral stimulation is near-background (or nonexistent), whereas the responses to contralateral and bilateral stimulation are localized and clearly evident. Close visual inspection of these surface plots also reveals that the magnitude of the SI response evoked by contralateral stimulation is greater than the response evoked by bilateral stimulation.

**Figure 3:**
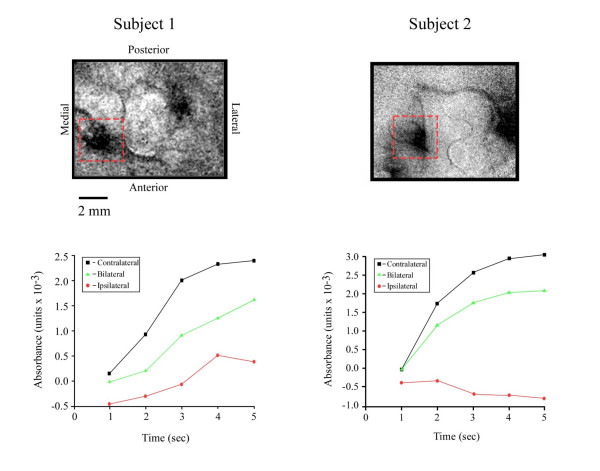
Graphs obtained from OIS data in SI cortical regions evoked by flutter stimulus on the central pad of 2 subjects. *Top*: Dashed box on OIS images indicates dimensions and orientation of region of interest (ROI) in SI used for analysis. Orientation and scales are identical in both subjects. Stimulus duration was 5 seconds; stimulus onset was at time 0 s. *Bottom: *Absorbance values within the ROI were averaged and plotted as a function of time for each stimulus condition. In both subjects contralateral stimulation evokes the largest change in absorbance, while ipsilateral stimulation evokes only a weak change in absorbance. Bilateral stimulation evokes an absorbance change that is less than that evoked by a contralateral stimulus.

Figure 3 enables one to directly compare the temporal sequence of the SI response to contralateral, ipsilateral, and bilateral central pad flutter. Each point in the plots in Figure 3 shows the average absorbance values within the ROI centered over SI at a particular time (frame) after stimulus onset. For both of the subjects that provided the data shown in Figure 3, the temporal sequences (one for each mode of stimulation) demonstrate that: (1) ipsilateral stimulation evokes little or no change in SI absorbance, (2) the magnitude of the absorbance change evoked in SI by bilateral stimulation is smaller than that evoked in the same SI region by contralateral stimulation, and (3) the discrepancy between the responses to bilateral *vs. *contralateral stimulation appears to increase with increasing time after onset of stimulation.

**Figure 4:**
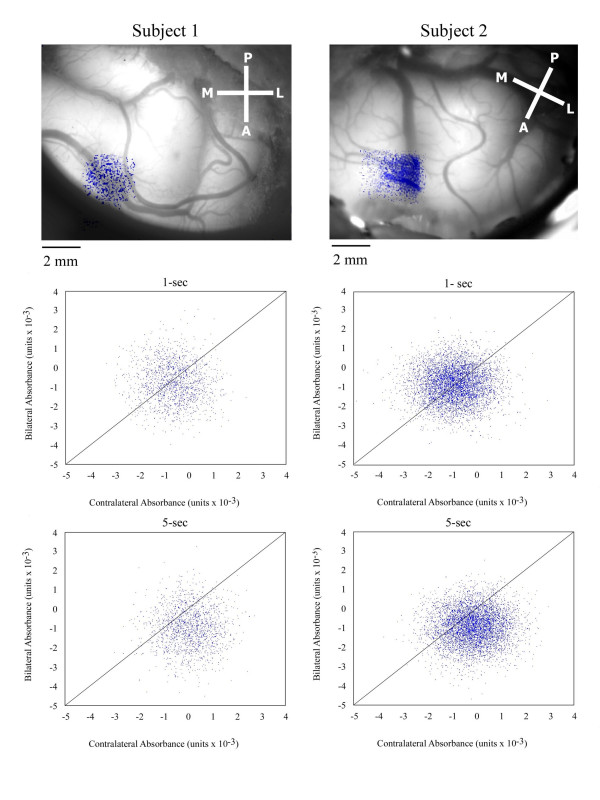
Scatter plots of contralateral vs. bilateral response of 2 subjects. For each plot, values of individual pixels are plotted as a function of the absorbance measured at that pixel evoked by the contralateral stimulus (horizontal axis) vs. the response measured at the same locus (or pixel) evoked by the bilateral stimulus (vertical axis). *Top panel: *cortical images taken with green filter, superimposed axes indicate anatomical orientation. Pixels superimposed on cortical image were selected using a threshold criteria (highest 5%) in response to both stimulus conditions and are represented by blue dots. Reference line, plotted at a slope of 1, indicates where pixels with equal values for both conditions lie.

Scatter plots were used to directly compare the response of SI to the different conditions of contralateral and bilateral stimulation. In each plot in Figure 4 the absorbance value obtained at each pixel under these two stimulus conditions is plotted against each other – i.e., the x-axis is the absorbance value evoked by the contralateral stimulus and the y-axis is the absorbance value evoked by the bilateral stimulus. The plots reveal that there is a difference in the population response of SI neurons to the contralateral *vs*. bilateral stimulus conditions. Additionally, the plots suggest that there is a time dependency in the development of the response to bilateral stimulation – that is, there is little difference between the responses to the two conditions at 1 sec after stimulus onset (at t = 1 sec the pixels are located symmetrically about the 45° line), yet at a later time (at t = 5 in each subject many of the pixels are shifted to a position below the reference line). Points located below (or to the right of) the reference line (slope = 1) represent pixels, or spatial locations in SI, where the response to contralateral stimulation is greater than the response to bilateral stimulation. It should be emphasized that this type of graphic does not reflect spatial differences in the responses to the different stimulus conditions, but rather, emphasizes whether or not different members of a set respond differently to the different stimulus conditions. Accordingly, the plots in Figure 4 indicate that with increasing time after onset of bilateral stimulation of the central pads (i.e., between 1 and 5 seconds after stimulus onset), absorbance at particular locations within the responding region of SI pixels reduces relative to the value achieved during contralateral stimulation. It also should be noted that the plots in Figure 4 show that not all spatial locations (pixels) in the responding region of SI exhibit this reduction in absorbance – that is, each plot indicates that at a minority of locations within the responding SI region bilateral stimulation evokes the same or even a greater response than the response evoked by contralateral stimulation.

**Figure 5:**
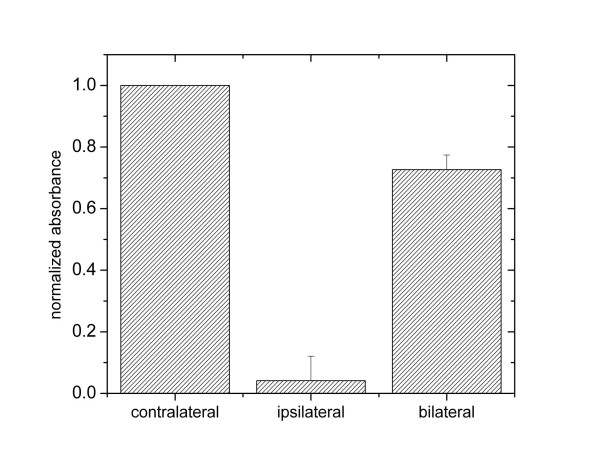
Comparison of averaged normalized absorbance values (n = 5) evoked under 3 different stimulus conditions. Values were normalized to those obtained from the contralateral stimulus condition (thus, standard error bar for the contralateral condition is zero) and were obtained at 5 sec after stimulus onset of a 5 sec flutter stimulus. The contralateral stimulus evoked the greatest change in absorbance, while the ipsilateral stimulus evoked the weakest. The bilateral stimulus evoked a cortical response that was less than the response evoked by the contralateral stimulus. Analysis of variance showed that, at a 99% confidence interval, the ratio of the bilateral response evoked to the contralateral response evoked in SI was between 0.51 and 0.92.

The across-subject consistency of the results was evaluated by determining the average absorbance values evoked by the 3 different stimulus conditions (with a 5 sec stimulus duration) for the 5 subjects at 5 sec after stimulus onset (Figure 5). Clearly, the contralateral stimulus condition evoked the largest average (across-subject) response, and therefore the values of the average (across-subject) absorbance increase obtained under the ipsilateral and the bilateral stimulus conditions were normalized to this absorbance value (thus standard error for the contralateral stimulus condition = 0). While the average response to ipsilateral stimulation was very weak or negligible, the average response evoked by bilateral stimulation was approximately 35% less than that evoked by contralateral stimulation. In each of the 5 subjects the response to bilateral stimulation was less than the response to contralateral stimulation. Statistical hypothesis testing was performed in order to determine if the average (across-subject) response evoked by bilateral stimulation was significantly less than the average response to contralateral stimulation (i.e., if the bilateral/contralateral response ratio was <1 at 5 sec after stimulus onset). Analysis of variance showed that the average bilateral/contralateral response ratio was between 0.51 and 0.92 in SI (p < 0.001; H_o _was that the ratio = 1).

### Comparisons between SI and SII responses

**Figure 6:**
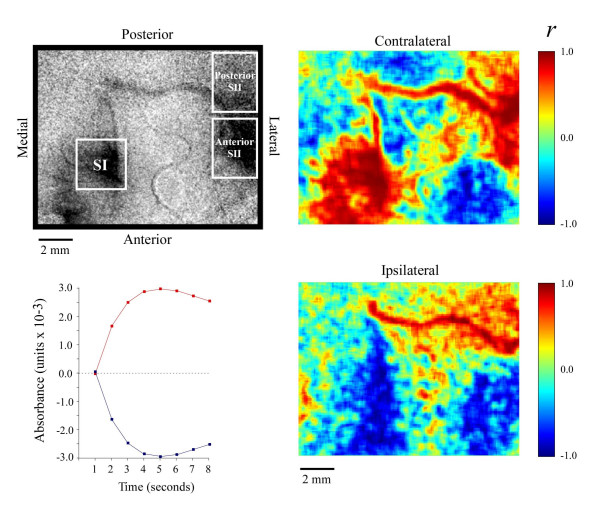
Correlation maps for contralateral and ipsilateral stimulus conditions in Subject #2. **Top Left**: Orientation of the maps. SI boxel indicates region of interest (ROI) used for obtaining averaged absorbance values. **Bottom Left**: Red line is graph of averaged time course of SI activity (from the ROI) used for generating correlation maps (from 1 to 8 seconds after stimulus onset). Blue line indicates the approximated time course of SI activity that would be negatively correlated. A correlation of +1 would correspond to the signal shown in dark red while a correlation of -1 would correspond to the signal in dark blue. **Top Right**: The correlation map generated from the contralateral response data shows positively correlated activity in SI as well as both regions of SII. **Bottom Right**: The correlation map generated from the ipsilateral response shows a positive correlation in only the posterior region of SII and a negative correlation in both the anterior region of SII and SI. Color bars indicate correlation coefficient values for each map.

Our previous study [4] characterized the SII response to the same contralateral, ipsilateral and bilateral stimulus conditions that we used to characterize the response of SI cortex in this report. The SII responses evoked by these different stimulus conditions led to the identification of two regions in SII arranged along the anterior and posterior axis of the anterior ectosylvian gyrus. In that study, identical contralateral vs. bilateral stimulus conditions at homotopic skin sites demonstrated that the response evoked by a bilateral stimulus was 35% smaller than that evoked by a contralateral stimulus in the anterior region of SII [4]. This correspondence between the effects of bilateral stimulation on SI and the posterior region of SII in the same hemisphere led us to evaluate the extent to which the changes in the activity of posterior SII and SI observed under this condition are inter-related. To investigate the relationship between the spatiotemporal patterns of absorbance between the different cortical regions – SI, anterior SII, and posterior SII – correlation analysis was performed on the data. Figure 6 shows the results obtained when this approach is used to evaluate the relationship between the responses to contralateral and ipsilateral stimulus conditions for an exemplary subject. For both correlation maps, a correlation value is obtained for each location (pixel) in the responding SI region by cross-correlating the time course of the absorbance changes at that location with a "standard" time course. In the case of Figure 6, the "standard" time course that was correlated with each pixel was obtained from SI activity evoked by contralateral stimulation. The red line in the graph inset of Figure 6 is the standard time course obtained from the average response evoked within the indicated SI boxel. The blue plot in the graph inset is simply the negative of the red and indicates the time course of pixels that are negatively correlated with the standard time course. In the contralateral stimulus condition, note that the activity in each of the 3 zones within the responding region (indicated by boxels at top left of Figure 6) is positively correlated to the time course of activity evoked in SI by a contralateral stimulus. For the ipsilateral stimulus condition, however, only the activity in the region of SII is positively correlated with the activity in the responding region of SI, whereas the activity in SI and the anterior SII regions is negatively correlated. The negative correlation indicates that the time course of stimulus-evoked activity in SI and anterior SII is trending in a direction opposite to that observed under the contralateral stimulus condition, whereas in posterior SII the activity modifies in a manner similar to that observed in the responding region of SI. The correlation maps obtained from each of the other experimental subjects indicated similar trends.

**Figure 7:**
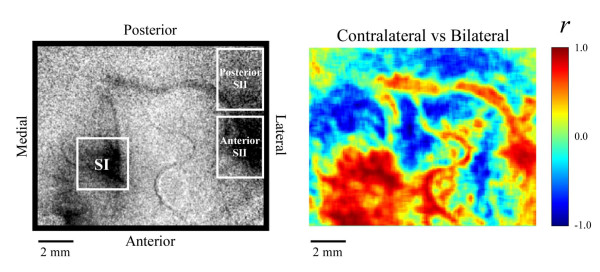
Cross-modal correlation map of Subject #2. Panel at top-left indicates orientation of the correlation map at right. The color bar shows correlation coefficient values used in the map. Correlation coefficients are determined by cross-correlation of time courses evoked at each spatial location to two different stimulus conditions. The map demonstrates that the responses to contralateral and bilateral stimulation are similar in SI and anterior SII, but that the different stimulus conditions evoke a weak or negative correlation in the posterior region of SII. Correlations were performed on data between 1 and 8 sec after stimulus onset.

A second method of correlation mapping was used to directly compare the responses evoked by contralateral *vs. *bilateral stimulation (a "cross-modal" correlation map). In this method, rather than correlating each pixel with a standard time course observed at one particular cortical region for the same stimulus condition, performs a cross correlates the time courses of absorbance values obtained at the same spatial location under different stimulus conditions. Thus, the correlation coefficient generated at each pixel location represents how similar (indicated by a positive correlation) or how different (indicated by a negative correlation) are the temporal responses of a cortical region recorded under 2 different stimulus conditions. The map in Figure 7 is indicative of the type result obtained by performing a "cross-modal" correlation (correlation of responses evoked by 2 different modes of stimulation) in all the subjects in which SI and SII was simultaneously imaged. Note that this type of correlation map shows that the responding region of SI and anterior SII (as defined by the boxels in the figure) have high values of correlation, whereas posterior SII shows only weak, or in some local regions, negative correlation. It should be pointed out that correlation values are an indication of how similar are the trends – in particular, the slopes of the time courses – of the activity of a specific region under the different modes of stimulation. Thus, although the activity levels of SI and anterior SII both undergo a reduction in magnitude (relative to the activity detected during contralateral stimulation) under the bilateral stimulus condition, the slopes of the time course are very similar – accordingly, the activity of the 2 regions is positively correlated under this condition. In posterior SII, on the other hand, response magnitude increases under the bilateral stimulus condition (relative to that obtained under the contralateral stimulus condition) – thus with bilateral stimulation the activity of this region is negatively correlated with the activity in SI and in anterior SII.

## Discussion

The findings of this study demonstrate that bilateral flutter stimulation of the central pads of the forepaws evokes an SI response significantly smaller than the response evoked when the stimulus is applied contralaterally. At the locus of the maximal OIS response evoked in the SI region by a contralateral stimulus, bilateral stimulation evoked a response that was, on average, 35% smaller than that evoked by the contralateral stimulus. Concurrently, at the locus of the maximal OIS response evoked by contralateral stimulation in anterior SII (reported in a previous study; [4]), bilateral stimulation evoked a response that was, on average, 35% lower than the activity evoked by a contralateral stimulus. The same conditions that led to the decrease in activity in SI and anterior SII also led to an increase in activity in posterior SII [4].

Although the neural mechanisms responsible for the above-described effect remains uncertain, some workers have reported that callosally-transmitted inputs exert modulatory, but inconsistent effects on SI neurons. Schnitzler et al [5], using MEG in humans, showed that tactile stimulation of one hand enhanced the response of ipsilateral primary somatosensory cortex (SI) to median nerve stimulation. Conversely, Korvenoja et al [6] reported that activation of SI (measured using MEG in humans) by electrical stimulation of the contralateral median nerve was suppressed during movement of the fingers of the ipsilateral hand. In addition, Hoechstetter et al. [7] described "interactions" in SII cortex (defined as a response that was less than a summation of the responses to independent ipsilateral and contralateral stimulation) during bilateral stimulation, but reported that the response in SI evoked by a contralateral stimulus was not altered by the addition of an ipsilateral stimulus. Shimojo et al [8] also found no difference in sources localized to SI evoked by unilateral versus bilateral stimulation. Most recently, Staines, et al, [9] reported data (fMRI) showing that SI activity evoked by passive bilateral stimulation is weaker than the SI activity evoked by passive unilateral stimulation. Other findings in the same report showed that the modulation of SI activity, particularly in the case of bilateral stimulation, was task-dependent. Others have shown that the activity evoked in SI by a unilateral stimulus can be modulated in a context-dependent manner, both in humans (e.g., [10,11]) and in other primates (e.g., [12-15]).

Although an influence mediated via direct callosal projections from ipsilateral SI to contralateral SI [2] should not be ruled out, the data presented in this study lead us to propose that SII is the major source of the modulated SI response to contralateral skin stimulation that is observed under a variety of stimulus conditions [16-19]. Whereas the available literature is contradictory [20], it is clear that SII in cats receives its principal input from the thalamus [21-25]. While some investigators have proposed that monkey SII occupies a higher position in the somatosensory information processing hierarchy than does SII in cat [20], others have warned that "these are basically descriptive schemes of connections that do not illuminate what features of somesthesis are selectively projected" and that "they also tend to ignore potential interdependence between SI and SII" [26]. The OIS observations in the present study obtained by simultaneously imaging the contralateral SI and SII in cats during ipsilateral stimulation of the central pad revealed that the time course of the optical response (increasing absorbance) evoked in posterior SII is negatively correlated with the time course of the optical signal in SI and anterior SII. Perhaps more significantly, the results obtained by correlation of the responses evoked under contralateral and bilateral stimulus conditions revealed a similar relationship between the cortical activity evoked in SI, anterior SII and posterior SII – in this case, the time course of the activities of SI and anterior SII are positively correlated under the bilateral stimulus condition, whereas under the same condition those activities are negatively or only weakly correlated with the activity in posterior SII. A plausible (but not necessarily the only) explanation for these outcomes is that the activity evoked in posterior SII exerts an inhibitory influence on SI via the extensive corticocortical connections known [27,28] to link topographically corresponding regions in SI and SII.

An extensive literature addresses the neuroanatomical routes by which information about the status of skin mechanoreceptors accesses SI and SII (see [27,28] for review). While the data presented in this paper neither extend or modify what is known about the routes by which information reaches SI and SII, they (along with the evidence presented in our previous paper – [4]) address the issue of whether, and to what extent, the responses of SI, anterior SII, and posterior SII in the same hemisphere are independent. The data presented here, in conjunction with evidence published previously [4], shows that nonnoxious mechanical skin stimulation evokes afferent activity that is conveyed via central somatosensory pathways to SII in both the contralateral and the ipsilateral hemispheres, and raises the possibility that unlike the activity evoked by contralateral flutter, the SII activity generated by ipsilateral skin stimulation may depress (perhaps via inhibitory processes mediated by corticocortical connections) the response of SI to ongoing skin stimulation. Previously, we postulated that SII activity levels modulated the SI response when the SII activity was evoked by vibratory stimuli [18]. That study showed that contralateral vibration increased SII activity levels which were correlated with decreases in SI vibration-evoked activity. The findings make it seem likely that, at least in cat, the degree to which SII activation modifies the SI response to skin stimulation depends on the attributes of the peripheral stimulus – that is, that SI activity is modified by SII activity in a stimulus-dependent manner.

## Conclusion

The responses evoked by contralateral, ipsilateral and bilateral flutter stimulation of the central pad of the cat forepaw indicate that the response of SI evoked by contralateral stimulation is reduced in the presence of an ipsilateral stimulus. Correlation analysis of the responses evoked by ipsilateral skin flutter stimulation showed that activity in posterior SII (the region of SII that maximally responds to an ipsilateral stimulus) is negatively correlated with the activity in SI. Additionally, correlation mapping of the results obtained from the contralateral vs. bilateral stimulus conditions demonstrated that the time course of the activities evoked in SI and anterior SII by these conditions are similar. The data lead to the proposal that increasing levels of activity in posterior SII evoked by an ipsilateral skin stimulus suppress/inhibit the responses normally evoked in both anterior SII and SI by contralateral stimulation.

## Methods

### Subjects & preparation

Adult cats (males and females; n = 5) were subjects. All surgical procedures were carried out under deep general anesthesia (1 – 4% halothane in a 50/50 mixture of oxygen and nitrous oxide). After induction of general anesthesia the trachea was intubated with a soft tube and a polyethylene cannula was inserted in the femoral vein to allow administration of drugs and fluids (5% dextrose and 0.9% NaCl). For each subject, a 1.5 cm diameter opening was made in the skull overlying somatosensory cortex, a chamber was mounted to the skull over the opening with dental acrylic, and the dura overlying anterior parietal cortex was incised and removed. Following the completion of the surgical procedures all wound margins were infiltrated with long-lasting local anesthetic, the skin and muscle incisions were closed with sutures, and each surgical site outside the recording chamber was covered with a bandage held in place by adhesive tape.

Subjects were immobilized with Norcuron and ventilated with a gas mixture (a 50/50 mix of oxygen and nitrous oxide; supplemented with 0.1 – 1.0% halothane when necessary) delivered via a positive pressure respirator 1–3 hours prior to the data acquisition phase of the OIS imaging experiments. Respirator rate and volume were adjusted to maintain end-tidal CO2 between 3.0 – 4.0%; EEG and autonomic signs (slow wave content; heart rate, etc.) were monitored and titrated (by adjustments in the anesthetic gas mixture) to maintain levels consistent with light general anesthesia. Rectal temperature was maintained (using a heating pad) at 37.5°C.

Euthanasia was achieved by intravenous injection of pentobarbital (45 mg/kg) and by intracardial perfusion with saline followed by fixative (10% formalin). Following perfusion fiducial marks were placed to guide removal, blocking, and subsequent histological sectioning of the cortical region studied. All procedures were reviewed and approved in advance by an institutional committee and are in full compliance with current NIH policy on animal welfare.

### Stimuli and stimulus protocols

Results were obtained during stimulation of the contralateral central pad of the forepaw and/or the ipsilateral central pad of the forepaw. The stimuli always consisted of sinusoidal vertical skin displacements (25 Hz, 400 microns, stimulus duration 5 sec, inter-stimulus interval 60 sec) and were applied using a pair of servocontrolled transducers (Cantek Enterprises, Canonsburg, PA) that is capable of delivering sinusoidal stimuli in the range of 1–250 Hz at amplitudes in the range of 0–1000 microns. The stimuli were delivered independently to the ipsilateral and contralateral skin sites, and also were applied simultaneously to both sites (bilateral stimulation). The stimulus probes were positioned 500 microns beyond the point at which skin contact was detected (via force transducer on the Cantek). The bilateral stimulus protocols reported in this paper were synchronized to start and stop at the same time. The contralateral, ipsilateral and bilateral stimuli were interleaved on a trial-by-trial basis. This approach was used to control for temporal changes in cortical "state" unrelated to stimulus conditions which, if unrecognized, might obscure or modify any differences between the optical responses evoked by the contralateral, ipsilateral and bilateral stimulus conditions.

### OIS imaging

Near-infrared (IR; 833 nm) OIS imaging was carried out using an oil-filled chamber capped with an optical window [29] Images of the exposed cortical surface were acquired 200 msec before stimulus onset ("reference" or "prestimulus" images) and continuously thereafter ("poststimulus" images; at a resolution of one image every 0.5 to 1.5 sec) for 15–20 sec following stimulus onset. Exposure time was 200 msec. Absorbance images were generated by subtracting each prestimulus (reference) image from its corresponding poststimulus image and subsequently dividing by the reference image. Averaged absorbance images typically show regions of both increased absorption of IR light and decreased absorption of light (to a depth of approximately 1400 microns) which have been shown to be accompanied by increases and decreases in neuronal activation, respectively [30-35].

### Correlation analysis

Correlation maps were constructed for comparison of spatio-temporal characteristics of the OIS response. One aspect of this method of analysis has been previously described in detail [18,29]. Briefly, the correlation maps in Figure 6 were constructed by choosing a reference region within the imaged field and computing the intensity correlation r_ij _between the absorbance value of each pixel (*i*, *j*) and the average absorbance value within the reference region over the time from stimulus onset to stimulus offset. The region selected as the reference was defined by a boxel (π mm^2 ^area) centered on the region of interest (ROI). Each pixel (*i*, *j*) on the correlation map is represented by a coefficient of determination *r*^2^_*ij *_(-1 <*r*^2 ^< 1; - 1 indicates negative correlation; + 1 indicates positive. The statistical significance of each of the correlations was tested with the standard t-test. A second type of correlation map, or cross-correlation map, was generated in a similar manner (such as that computed in Figure 7). In this method, rather than correlating each pixel with the time course observed at one particular cortical region for the same stimulus condition, cross correlation was performed between the time courses of absorbance values obtained at the same spatial location (*i*, *j*) for different stimulus conditions. Thus, each coefficient of determination *r*^2^_*ij *_represents how similar (positive correlation) or how different (negative correlation) the response in a region is to a change in stimulus condition.

### Histological procedures/identification of cytoarchitectural boundaries

At the conclusion of the experiment, the imaged cortical region was removed immediately following intracardial perfusion with saline and fixative. The region then was blocked, postfixed, cryoprotected, frozen, sectioned serially at 30 μm, and the sections stained with cresyl fast violet. The boundaries between adjacent cytoarchitectonic areas were identified by scanning individual sagittal sections separated by no more than 300 mm and were plotted at high resolution using a microscope with a drawing tube attachment. The resulting plots then were used to reconstruct a two-dimensional surface map of the cytoarchitectonic boundaries within the region studied with optical and neurophysiological recording methods. The locations of microelectrode tracks and electrolytic lesions evident in the histological sections were projected radially to the pial surface and transferred to the map of cytoarchitectonic boundaries reconstructed from the same sections. As the final step, the cytoarchitectonic boundaries (along with the locations of microelectrode tracks and lesions whenever present) identified in each brain were mapped onto the images of the stimulus-evoked intrinsic signal obtained from the same subject, using fiducial points (made by postmortem applications of india ink or needle stabs) as well as morphological landmarks (e.g., blood vessels and sulci evident both in the optical images and in histological sections). Locations of cytoarchitectonic boundaries were identified using established criteria [36-38].

## Authors' contributions

BW and OF participated in the design of the experiments, the data collection, and drafting of the manuscript. SS and JC made significant contributions to the data collection and the analysis of the data. MT played a major role in all aspects of the development of the manuscript.
